# Matron's in Council: IV. The Love and Sympathy of a Noble Woman

**Published:** 1917-08-25

**Authors:** 


### THE MATRONS' AND SISTERS' DEPARTMENT.

### MATRONS IN COUNCIL.

### IV. The Love and Sympathy of a Noble Woman.

The article in The Hospital of August 4 has aroused much interest in the nursing world, and no matron of a hospital can read it without asking herself if she is living up to the ideal she set for herself when she accepted office. It is very true that the tone of a whole hospital is taken from the matron, and it is to be feared that many of us are too autocratic, and unable (or unwilling) to see things from any point of view but our own. The one quality that is essential for every hospital worker is sympathy. No nurse is really good unless she has sympathy with her patient; no sister of a ward is a good sister unless she has sympathy not only with her patients, but with her nurses; and no matron is a good matron unless her sympathies are wide enough to embrace all working with her and under her charge. She should be able to put herself in the place of the newest probationer and understand her needs and her failings, just as well as she should be able to sympathise with the trials and worries of the ward sister. In any difficulty arising between a patient and a nurse sympathy will help her to see both sides of the case, and her judgment will be unbiassed by professional predilection. In every large institution there is bound to be friction; a large number of people of different dispositions and characters living in constant touch with each other must sometimes clash, and it is the part of the matron to apply the "oiled feather." It is she who should inculcate the virtues of loving-kindness and courtesy, of consideration for others in word and action. In the present day love of service is a good old-fashioned virtue which, I am afraid, is sometimes relegated to the scrap-heap; but, in my opinion, without the love of service a nurse will be but a machine, well trained possibly, but still a machine.

The greatest difficulty with which a matron has to contend now is that hospitals are so large that most of her day is taken up with routine work, and there is a great fear that she may become nothing more than an office machine. She has so much to do, so much to control, that it leaves little time for studying the different characters and dispositions of her nurses; when her correspondence is finished, her rounds made, and all the thousand and one details of hospital routine finished, she is often too tired to give more than a perfunctory attention to the personal side of her work.

With regard to examinations, I think theoretical knowledge is necessary for every nurse, provided it be thorough and that it helps her practical work. It is not necessary for a nurse to be too theoretical; she is "the hands" of the doctor, and her duty is to learn to obey his orders implicitly, to train her observation, so that she may be able to give an accurate report of her patient when he pays his visits, and to learn to give it with accurate conciseness. All this should be learnt in the course of training, and I, personally, think that it is far more necessary for a nurse to be practical, conscientious, reliable, observant, tactful, sympathetic, and tender than that she should be brilliant theoretically if we cannot have both. Above all, let nurses beware of acquiring that smattering of knowledge which is useless, and, in their profession, dangerous.

With regard to the pay of nurses, it seems to me that the probationer has nothing of which to complain. There is no other profession in which the student is kept from the day in which she enters it. For the first year of training all a probationer has to provide is her own pocket-money and any private clothes she may choose to buy. In the art world many years have to be devoted to study before there is the slightest chance of remuneration; in the business world no girl expects to earn enough to keep and clothe herself while learning her trade. In a hospital, however, there are few "plums," and I think the pay of a ward sister might well be increased. She has given many years of her life to her profession and has great responsibility, and the pay she receives does not permit her to save much for old age.

In conclusion, I would urge most strongly that the tendency for hospitals to become over-grown, machine-like institutions should be combated with all our might, and that the matron should not be so over-loaded with other duties that her paramount duty of caring for the welfare of her nurses has rather to be put on one side or left to others. Let us not forget that the vital spark of every good hospital is loving-kindness, and let us try to unite the love of service and sympathy with mankind, which inspired Florence Nightingale, to the modern ideal of efficiency. There is no reason why the nurse who smooths a patient's pillow tenderly should not be as efficient and as thorough as a hard, well-drilled automaton, and surely she will send that patient out to the world again a better citizen for having felt the love and sympathy of a noble woman.

☛ Previous Articles appeared in The Hospital of August 4, 11, and 18.

